# VPBR: An Automatic and Low-Cost Vision-Based Biophysical Properties Recognition Pipeline for Pumpkin

**DOI:** 10.3390/plants12142647

**Published:** 2023-07-14

**Authors:** L. Minh Dang, Muhammad Nadeem, Tan N. Nguyen, Han Yong Park, O New Lee, Hyoung-Kyu Song, Hyeonjoon Moon

**Affiliations:** 1 Department of Information and Communication Engineering, and Convergence Engineering for Intelligent Drone, Sejong University, Seoul 05006, Republic of Korea; minhdl@sejong.ac.kr (L.M.D.); songhk@sejong.ac.kr (H.-K.S.); 2 Department of Computer Science and Engineering, Sejong University, Seoul 05006, Republic of Korea; 22110104@sju.ac.kr; 3 Department of Architectural Engineering, Sejong University, Seoul 05006, Republic of Korea; tnnguyen@sejong.ac.kr; 4 Department of Bioresource Engineering, Sejong University, Seoul 05006, Republic of Korea; hypark@sejong.ac.kr (H.Y.P.);

**Keywords:** pumpkin, deep learning, measurement, segmentation, biophysical properties

## Abstract

Pumpkins are a nutritious and globally enjoyed fruit for their rich and earthy flavor. The biophysical properties of pumpkins play an important role in determining their yield. However, manual in-field techniques for monitoring these properties can be time-consuming and labor-intensive. To address this, this research introduces a novel approach that feeds high-resolution pumpkin images to train a mathematical model to automate the measurement of each pumpkin’s biophysical properties. Color correction was performed on the dataset using a color-checker panel to minimize the impact of varying light conditions on the RGB images. A segmentation model was then trained to effectively recognize two fundamental components of each pumpkin: the fruit and vine. Real-life measurements of various biophysical properties, including fruit length, fruit width, stem length, stem width and fruit peel color, were computed and compared with manual measurements. The experimental results on 10 different pumpkin samples revealed that the framework obtained a small average mean absolute percentage error (MAPE) of 2.5% compared to the manual method, highlighting the potential of this approach as a faster and more efficient alternative to conventional techniques for monitoring the biophysical properties of pumpkins.

## 1. Introduction

Pumpkin, a popular winter squash native to North America, has gained worldwide cultivation. Belonging to the Cucurbitaceae family alongside various squash types, cucumbers, and melons [1], pumpkins are renowned for their large, round, or oblong shape and thick, hard rind. While commonly orange, their rind can also appear in shades of green, yellow, or white. Notably, their vibrant orange flesh is widely utilized in fall season delicacies such as pies, soups, and other dishes. Pumpkins are cultivated and consumed in various parts of the world, including Asia, Europe, and North America. China stands out as a significant presence in pumpkin production, being one of the leading producers in Asia and globally [2]. Additionally, various Asian countries, such as South Korea, have a rich tradition of incorporating pumpkins, known as “hobak” in Korean [3], into their cuisine. Traditional Korean dishes often feature pumpkins in soups, stews, and porridges as a side dish or snack. It is worth noting that pumpkins grown in Korea are distinct; they are characterized by their smaller size, heightened sweetness, denser texture, and nuttier flavor, setting them apart from varieties found in other regions of the world.

The regular monitoring of pumpkin biophysical properties is vital throughout the growing season to assess crop conditions, model crop growth, and predict potential yields [4]. Understanding and enhancing the pumpkin industry, from breeding and production to marketing and consumption, heavily relies on the examination of these properties. The biophysical properties of pumpkins offer valuable insights into their genetics and physical characteristics, empowering plant breeders to develop new varieties with desirable properties, mitigate disease risks, boost yield, and enhance marketability. Moreover, these properties serve as essential indicators for farmers and consumers, providing information regarding pumpkin maturity, quality, and adherence to standards of size, shape, and color [5]. By comprehending and leveraging these properties, stakeholders can ensure optimal pumpkin production and consumption while meeting the expectations of diverse markets.

Previously, the measurement of biophysical properties heavily relied on manual investigations, which presented certain limitations. For instance, Nankar et al. conducted a manual evaluation of various phenotypic and biochemical properties, including plant height, fruit weight, color, firmness, and virus resistance [6], to underscore the significance of phenotypic traits in characterizing and selecting plant accessions for breeding and crop improvement. Similarly, Öztürk et al., assessed 11 pumpkin genotypes [7], examining morphological characteristics like fruit shape, size, color, seed color, and seed size. Additionally, they utilized molecular markers, such as random amplified polymorphic DNA (RAPD) and inter-simple sequence repeat (ISSR) markers, to analyze the genetic diversity and relationships among the pumpkin genotypes. However, relying solely on the manual measurement of phenotypic traits can be a time-consuming and labor-intensive process, particularly when dealing with a substantial number of samples. This approach can result in high costs, slow data acquisition, and the potential for errors or inconsistencies due to human fatigue or subjectivity. Moreover, manual methods may lack scalability and efficiency when it comes to large-scale phenotypic characterization, especially for complex properties that necessitate multiple measurements or observations.

To address these challenges effectively, an increasing demand for advanced and automated techniques in measuring biophysical properties for pumpkin research has emerged. Such techniques offer streamlined data collection, reduced human effort, improved accuracy, and enhanced scalability for phenotypic characterization. Incorporating automated methods and technologies enables researchers to expedite the evaluation process, obtain more robust data, and facilitate an efficient analysis of complex properties. Recent advancements in computer vision (CV) and deep learning (DL) technologies [8,9] have made it possible to automate the measurement of biophysical properties, significantly reducing the time and effort required for assessment. CV techniques allow pumpkins to be captured through cameras, and the resulting images can be analyzed using DL algorithms that accurately measure biophysical properties like width, length, and color.

For example, Wittstruck et al. utilized RGB imagery acquired from a commercial unmanned aerial vehicle (UAV) at different stages of pumpkin growth [10]. They processed the images using CV techniques to automatically extract information related to plant height, canopy width, and fruit count. The authors demonstrated a strong correlation between the UAV-based RGB imagery and ground-based measurements. Dang et al. showcased the potential of UAV-based RGB imagery in reducing labor and time for radish disease monitoring, making it a cost-effective and efficient tool for precision agriculture [11]. Ropelewska et al. presented a non-destructive method for classifying different pumpkin cultivars based on flesh characteristics [12], employing various CV methods. The experimental results showed an accurate classification of pumpkin cultivars based on flesh characteristics with over 90% accuracy. Notably, color and texture were identified as crucial features for classification, as they corresponded to the distribution and quantity of carotenoids and fibers in pumpkin flesh. Furthermore, Longchamps et al. emphasized the significance of yield-sensing technologies in horticulture, providing a comprehensive overview of the technologies developed and tested for perennial and annual horticultural crops [13]. This information is valuable for growers and researchers seeking to enhance their understanding of yield-sensing technologies and their potential applications in horticulture. Compared to manual approaches, CV-based methods offer non-destructive measurements, enabling repeated assessments of the same sample without damage or alteration. This facilitates high-throughput data acquisition and analysis [14,15], leading to a more comprehensive and accurate characterization of phenotypic traits.

According to a review conducted by Li et al. [16], previous studies have explored the manual measurement of phenotypic traits and genetic diversity in various plants and crops; however, they often face challenges in terms of scalability and efficiency, particularly when it comes to large-scale phenotypic measurement or complex properties requiring multiple measurements or observations. The main objective of this study is to establish a framework that uses smartphone imagery to extract the precise biophysical properties of pumpkins. By implementing the framework introduced in this study, it becomes feasible to automatically evaluate the growth status of pumpkins and integrate complex biophysical properties that traditional breeding methods may not accomplish, thereby enabling the development of new varieties with enhanced speed and accuracy. This approach allows for the development of new pumpkin varieties with enhanced speed and accuracy. Moreover, the study specifically focuses on pumpkins, highlighting their unique characteristics, such as the distinctive properties of Korean pumpkins mentioned earlier. By considering the specific context of pumpkin cultivation and consumption, insights that are tailored to the pumpkin industry and its stakeholders were provided. The specific objectives of this study include: (i) introducing a pumpkin component segmentation model based on SOLOv2 to effectively recognize relevant components, (ii) automatically measuring seven distinct biophysical properties of pumpkins using the predicted masks, and (iii) estimating the real-life values corresponding to the extracted biophysical properties.

The remaining manuscript contents are organized as follows: Various experiments are presented in Section 2 to evaluate various aspects of the proposed framework. In Section 3, the key findings and implications of the study are discussed. Section 4 describes the pumpkin dataset for training the segmentation model. Section 5 discusses the fundamental components of the proposed framework used to measure a pumpkin’s biophysical properties automatically. Finally, Section 6 provides a summary of the findings and suggests potential areas for future research.

## 2. Results

This section provides a comprehensive evaluation of the proposed model through a series of experiments. Section 2.1 demonstrates the effectiveness of the color calibration process on outdoor images, showcasing its impact on improving accuracy. Section 2.2 thoroughly assesses different aspects of the SOLOv2 model trained on the color-calibrated pumpkin dataset, offering insights into its performance and capabilities. Finally, in Section 2.3, we compare the results of the automated biophysical properties measurement with the actual measurements, providing a comprehensive analysis of the model’s performance and its ability to accurately estimate the properties.

### 2.1. Color Calibration

Color correction is a critical image processing technique that enhances the accuracy, natural appearance, and visual appeal of colors in an image, making them appear true to real life. This process is particularly crucial for accurately extracting the color properties of pumpkins. Ideally, under controlled conditions, the RGB values of color checkerboard chips from the reference image should exhibit a linear relationship with those from the collected images captured outdoors [17]. However, due to the constantly changing outdoor lighting conditions, the color chips in these images may deviate from the expected linear trend.

**Figure 1:**
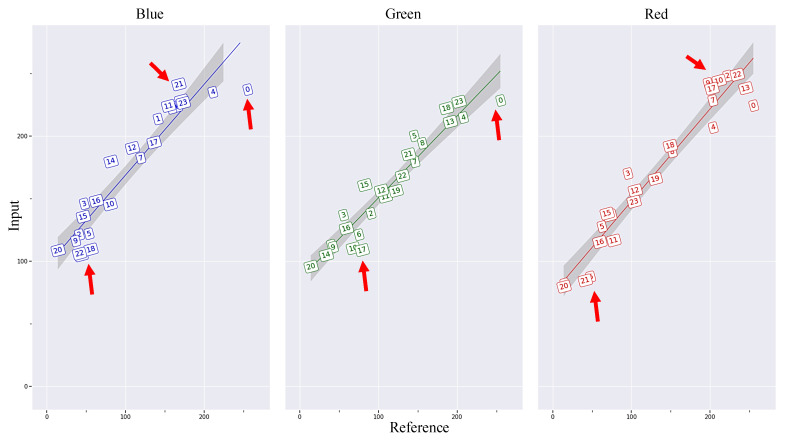
Comparison of the R, G, B color values of various color chips from the color checker between an input image and the reference image. Note: The red arrows point to sample problematic chips in the input image deviate from the linear trend line.

Figure 1 provides a visual comparison between the reference image and the collected image for color-checking purposes. The matrix displayed in the figure represents the average red, green, and blue channel values for each color chip on the color checkerboard in both images. The presence of red arrows indicates specific problematic chips in the source image that do not follow the linear trend line for the red, green, and blue color channels. This visual representation strongly emphasizes the significance of the color correction process in achieving accurate color extraction and analysis.

**Figure 2:**
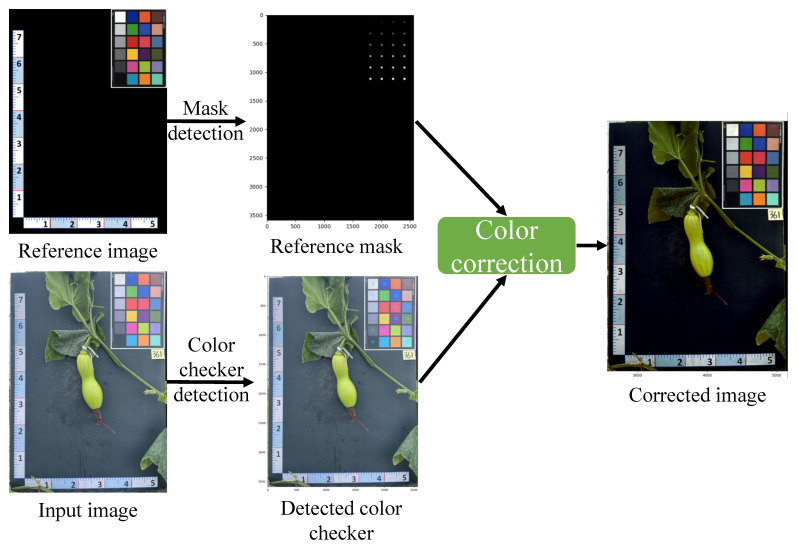
Example of the color calibration process, which takes the source image and target image as input and outputs the calibrated image.

The image depicted in Figure 2 shows the outcomes achieved by applying color correction to an input image utilizing a reference image. The procedure involved extracting the reference mask and input mask, which accurately identified the color checker’s location in both images. Next, the color space was obtained from both the reference and input images by detecting the color checker. Finally, the color space of the input image was transformed to align with the preferred color space of the reference image. The corrected input image exemplifies the enhanced accuracy and consistency of colors when compared to the original input image.

### 2.2. SOLOv2 Performance Evaluations

**Figure 3:**
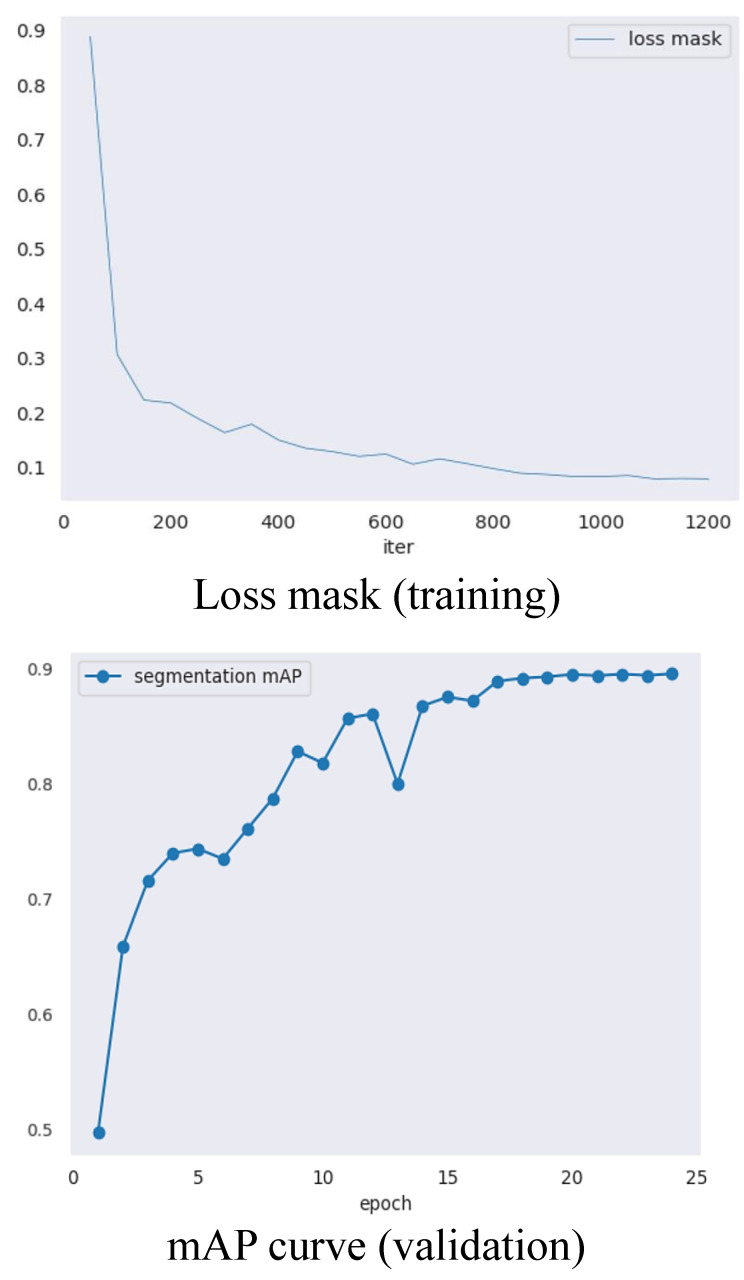
Training loss and validation mAP curves of the SOLOv2 model using the ResNet101 + DCN backbone network.

After applying color correction, the processed images were used to train and evaluate the SOLOv2 model with a ResNet101 backbone. The training loss mask and validation mAP results are illustrated in Figure 3, revealing the effectiveness of the training process and the stable convergence of the SOLOv2 model. The training loss mask showed a significant decrease, reaching approximately 0.23 after 200 iterations, and eventually converged to a stable loss of around 0.1 by the end of the training process (iteration 1200). The validation mAP steadily increased, surpassing 0.6 after the 10th epoch and peaking at 0.88 during the 25th epoch. These results demonstrate the model’s robust segmentation capabilities and its ability to consistently provide accurate segmentation for both pumpkin fruit and vine components, indicating strong generalization capabilities.

To assess the segmentation performance of the SOLOv2 model, we performed a comprehensive comparison with four other state-of-the-art segmentation models: BlendMask [18], HTC [19], MS R-CNN [20], and Mask-RCNN [21]. Quantitative evaluation metrics including mask AP and inference speed were computed. The results of the evaluation are summarized in Table 1, with the best-performing values for each metric on the collected pumpkin segmentation dataset highlighted in bold font.

Table 1 demonstrates the segmentation performance of different algorithms, where both SOLOv2 and BlendMask stand out with impressive mask AP scores of 88% and 85.9%, respectively. These results highlight the superior performance of these algorithms in accurately segmenting pumpkin samples. When considering inference speed, SOLOv2 also demonstrated exceptional performance by achieving a processing speed of 19.4 FPS, which is nearly double the speed of Mask-RCNN at 11.2 FPS. On the other hand, HTC exhibited a significantly slower inference speed of 6 FPS. Taking into account both mask AP and inference speed, SOLOv2 emerges as the top-performing algorithm among the evaluated models. It not only achieves the highest mask AP score but also boasts the fastest inference speed, making it a compelling choice for pumpkin instance segmentation.

**Figure 4:**
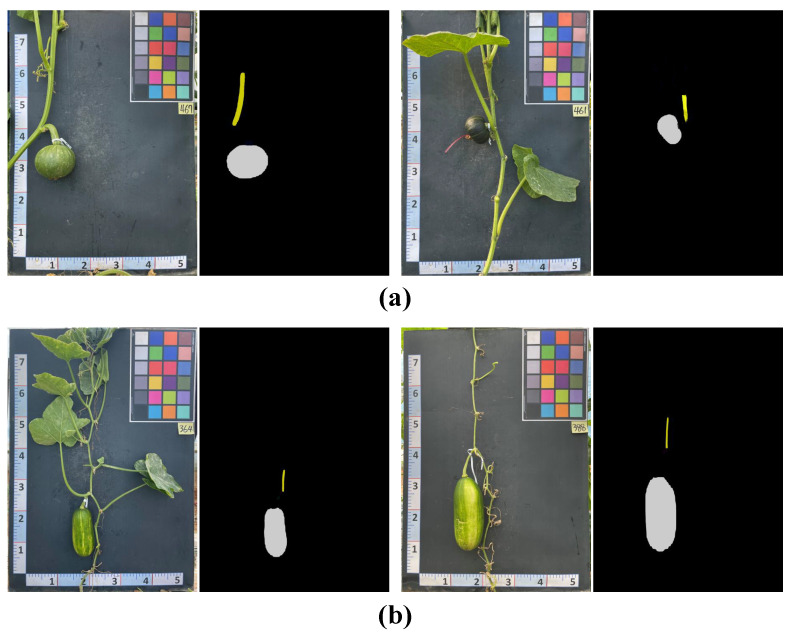
Four examples of the pumpkin segmentation results using the SOLOv2 model. Note: (**a**) is the results for two *Cucurbita maxima* samples and (**b**) is the results for two *Cucurbita moschata* samples.

Figure 4 demonstrates four examples of predicted masks generated by the SOLOv2 model, consisting of two samples for *Cucurbita maxima* (*C. maxima*) and two for *Cucurbita moschata C. moschata* pumpkins. Despite the similarity in color between the vine and fruit components, the model accurately distinguished and localized them. Notably, it precisely identified the vine sections closest to the fruit stem while avoiding false detections of vine parts farther away. These outcomes emphasize the robustness and effectiveness of the model in accurately segmenting the various components of pumpkins.

By utilizing SOLOv2, the framework was able to achieve precise segmentation, enabling the extraction of essential biophysical properties. The selection of SOLOv2 ensures that the proposed system delivers accurate and reliable measurements, enhancing the overall quality of the pumpkin biophysical property recognition.

### 2.3. Biophysical Properties Measurement

Table 2 provides a comprehensive comparison between the GT and predicted measurements of various biophysical properties for 10 pumpkin samples (S1 to S10) using the proposed framework. The measured biophysical properties include FL, FW, VL, VW, and FPC. The table is divided into three main sections. The first section displays the GT values for each sample and biophysical property, providing a reference for the actual measurements. The second section presents the predicted values obtained through the proposed framework, showing the model’s prediction of the biophysical properties for each sample. Finally, the third section quantifies the accuracy, MAE, and MAPE of the predictions, evaluating the model’s performance for each sample and biophysical property.

The GT row provides the actual measurements of the biophysical properties for the ten pumpkin samples. These values are obtained through manual measurements of the pumpkins using a tape measure. The tape measure ensured precise and consistent measurements across different parts of the pumpkin. The range of FL spans from 6.2 mm (sample S8) to 27.8 mm (sample S2), while FW ranges from 8.1 mm (sample S7) to 12.9 mm (sample S4). VL varies from 6.5 mm (sample S10) to 16.2 mm (sample S3), and VW ranges from 0.67 mm (sample S6) to 1.8 mm (samples S5 and S9). The FPC is categorized as G, OR, and LG, with all samples sharing the same color except for samples S6 and S7, which exhibit an LG color.

The prediction row represents the biophysical properties’ measurements predicted by the framework for the 10 pumpkin samples. These predicted values are compared to the GT values to determine the accuracy of the predictions. The accuracy is evaluated using the formula: taking the absolute difference between the predicted and GT values, dividing it by the GT value, and multiplying the result by 100. The values in this row indicate a high degree of proximity between the predicted and GT values, demonstrating the accuracy of the model in measuring the biophysical properties of the pumpkins. The accuracy values are reported in the last row, ranging from 82.9% (sample S4) to 100% (samples S3, S5, and S10), with an overall accuracy of 96.1%. These results highlight the model’s capability to accurately measure the biophysical properties of pumpkins and its potential for broader applications in measuring properties of other vegetables.

Furthermore, the effectiveness and reliability of the proposed framework is reinforced by the low average MAE value of 0.87 and average MAPE value of 4.07% over the 10 samples. These metrics provide additional evidence for the effectiveness and reliability of the framework in accurately measuring biophysical properties. The precise measurements of biophysical properties achieved by the framework underscore its value in facilitating plant breeding programs and genetic studies, as it enables a dependable detection and quantification of crucial biophysical properties.

## 3. Discussion

Previous research has been predominantly based on manual approaches to compute the biophysical properties, which are tedious and susceptible to errors. This study overcame these drawbacks by introducing a vision-based system for accurately measuring the biophysical properties of pumpkins. The primary finding of our study demonstrated that the automated framework precisely measured the biophysical properties of pumpkin fruits and vines. In a wider context, prior studies have reported biophysical properties measurements in pixels, which can pose challenges for end-users in terms of interpretation [6,22]. This research successfully solved this problem by precisely converting pixel values into real-world measurements through the recognition of a ruler using a straightforward image processing pipeline (Section 5.1.4). This approach not only simplifies the result interpretation but also enables the establishment of a comprehensive database of phenotypic traits for pumpkins.

In addition to the main contribution of biophysical properties measurement in real-life units, this research suggests several innovative schemes that make significant contributions to the field of phenotyping. Firstly, many studies have pointed out the importance of the pre-processing module, particularly color correction [23,24] for plant biophysical properties measurement. We stressed the importance of this module for the images collected outdoors. This module, described in Section 5.1.2, implements color calibration to correct variations, which is particularly crucial given the potential impact of inaccurate color representation on biophysical properties like FPC. Even though this module requires more computational resources and time, it can be selectively enabled or disabled based on the specific requirements of the application.

Furthermore, previous research has often relied on contour detection [25] or bounding box/mask methods [26] to measure the width and length traits of fruits. However, these approaches are not accurate for fruits and plants with irregular shapes. In this study, we propose a novel method for computing the length property based on the skeletonization algorithm (Section 5.1.4). The length can be accurately determined by extracting the fruit’s skeleton, making this approach suitable for various fruits and plants with irregular shapes. Additionally, an affine transformation method was employed to precisely compute the width trait, considering any asymmetry in the fruit’s placement during data collection. The experimental results demonstrate the effectiveness of the proposed width and length measurement approach in this study with an average MAPE of 2.5% compared to manual measurements.

When considering the impact of our proposed methodology on breeders, it becomes evident that the automated computation of biophysical properties in real-life measurements provided by the proposed framework has a profound effect. It diminishes the need for manual measurements, which are both error-prone and time-consuming. By automating the biophysical properties measurement process, breeders are able to save valuable time and resources, thus enabling a more efficient analysis of larger populations of pumpkins. The availability of accurate and comprehensive phenotypic data further empowers breeders in their selection and breeding procedures, ultimately resulting in enhanced crop yield, improved quality, and overall advancements in breeding progress.

## 4. Materials

The primary objective of this dataset was to facilitate the automated measurement of various biophysical characteristics of pumpkins. The data were collected from two pumpkin greenhouses situated in Gyeonggi-do, Korea, spanning from September 2022 to November 2022. Each greenhouse had dimensions of 6.5 m (width) × 65 m (length) × 3.5 m (height). Stringent measures were established to ensure all collected images are uniformly captured. These measures involve careful control of the pumpkin fields through the implementation of drip irrigation. The irrigation system utilizes a nutrient solution comprising vital compounds like nitrogen, potassium, and phosphorus. The primary objective is to mitigate the occurrence of abiotic stresses, including drought and nutrient deficiencies, while concurrently minimizing the risk of diseases and pests. Furthermore, daily field inspections were carried out by farmers or specialists to proactively avoid the emergence of diseases, pests, or abiotic stresses.

Specifically, two predetermined pumpkin cultivars, namely *Cucurbita moschata* (*C. moschata*) and *Cucurbita maxima* (*C. maxima*) from the Cucurbitaceae family, were cultivated and monitored for the purpose of analysis. *C. moschata* exhibits a distinctive long, cylindrical, or oblong shape with slightly curved or crooked necks. Its fruit can reach lengths of 60–90 cm and have a diameter ranging from 10 to 30 cm. The skin of *C. moschata* pumpkins is typically tan or orange, and it can either be smooth or display slight ribbing. In contrast, *C. maxima* showcases a round or oval shape. The skin can be smooth or ribbed, and it displays a captivating range of colors, including orange, yellow, green, or white. The size of *C. maxima* pumpkins can vary significantly, ranging from small pie pumpkins to giant varieties, adding to their visual diversity.

**Figure 5:**
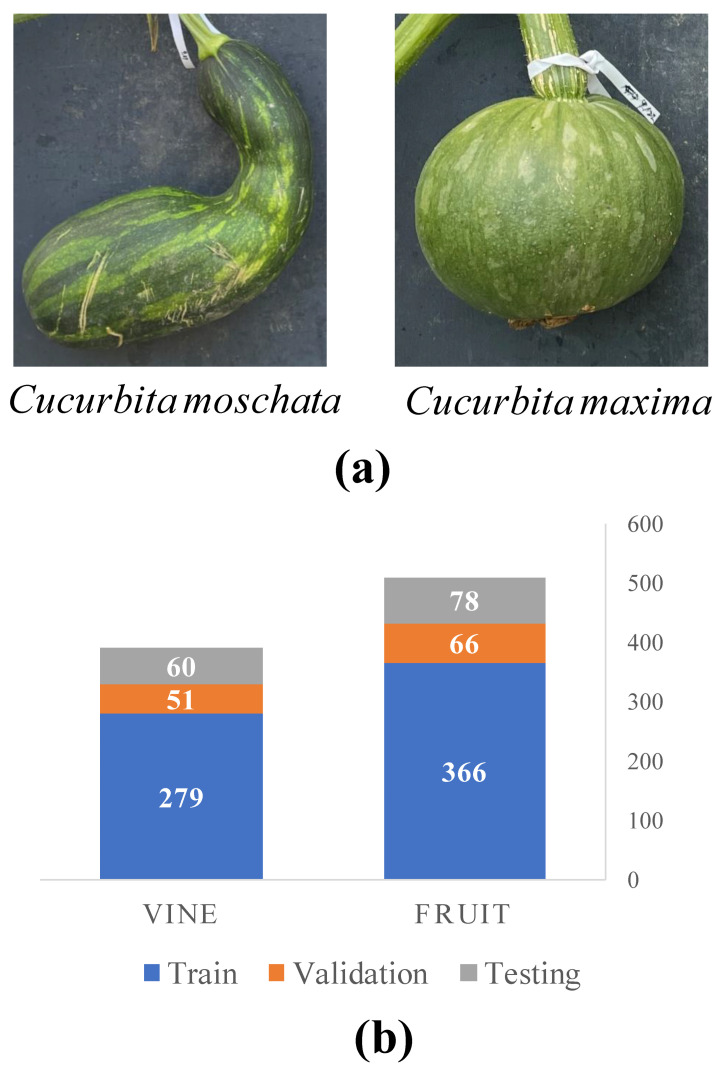
Sample images representing two different pumpkin cultivars in this study along with a description of the number of training and testing images collected for the vine and fruit components. Note: (**a**) shows sample images for the two pumpkin cultivars. (**b**) describe the collected segmentation dataset.

Data collection was conducted using a Samsung Galaxy S22 smartphone equipped with a rear camera that has impressive specifications, including a high resolution of 50 megapixels, an aperture of f/1.8, and advanced autofocus capabilities [27]. Each image has a consistent size of 3000×4000 pixels. The collection process took place within a specific one-hour time frame from 11 a.m. to 12 p.m., which coincided with the solar noon period. To guarantee uniform lighting conditions and minimize variations between images, additional attention was given to avoiding instances where clouds partially obstructed the sun. Additionally, an X-rite 4×6 color checkerboard containing 24 colors was employed [28]. This color checkerboard serves as a reference for image calibration, facilitating accurate color representation and enabling precise calibration during subsequent analysis, thereby enhancing the overall accuracy of the collected data. Sample images depicting the two pumpkin cultivars can be observed in Figure 5.

In order to achieve uniformed conditions for images captured using the smartphone, a tripod was utilized to ensure a consistent distance and angle between the camera and the test bed. The tripod was firmly positioned at the base of the test bed, securely holding the smartphone camera. By adhering to this setup throughout the image capture process, the tripod served as a reference point for both distance and angle. This approach allows images to be captured under uniformed settings, as the camera and test bed remained consistently aligned. As a result, this method substantially minimized the variability and enhanced the dependability of the subsequent analysis conducted on the dataset.

This study focuses on measuring the biophysical properties of both the pumpkin vine (the portion nearest to the stem) and the fruit. The pumpkin fruit holds significant importance due to its biophysical properties, including the size, shape, and color, which are important for breeders and growers in developing new cultivars that are more productive, nutritious, and appealing to consumers. On the other hand, the pumpkin vine serves as the structural foundation of the pumpkin crop, playing a critical role in essential functions such as nutrient and water uptake, photosynthesis, and providing support for the fruit. Understanding the biophysical properties of the pumpkin vine, such as vine length and diameter, is crucial for optimizing pumpkin plant growth and productivity. Furthermore, this knowledge aids in formulating effective management strategies to combat pests and diseases that can cause damage to the vine, resulting in reduced fruit yield. As depicted in Figure 5, a total of 900 images were collected and annotated. This dataset comprises 390 images for the vine class and 510 images for the fruit class. The distribution of training, validation, and testing images is summarized in Figure 5.

## 5. Methods

### 5.1. VPBR

**Figure 6:**
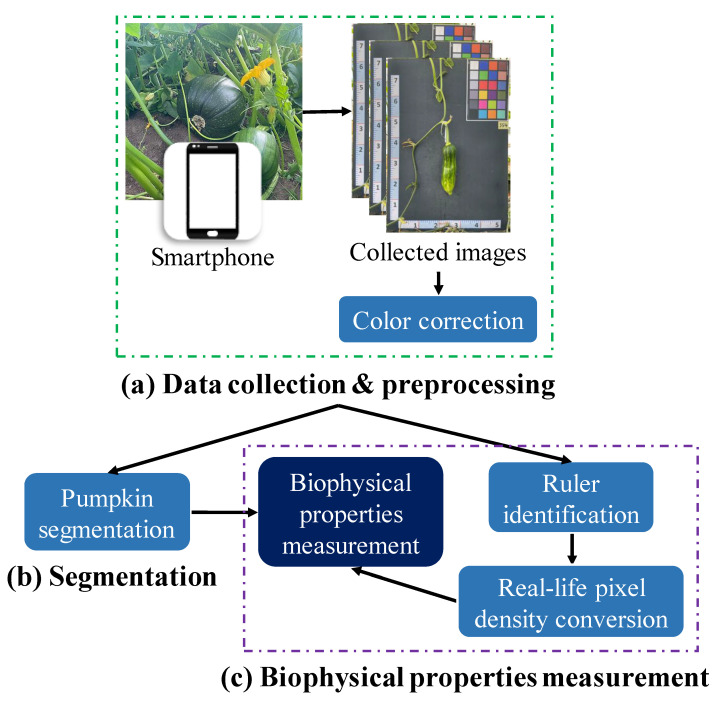
Illustration of the proposed biophysical properties measurement framework for pumpkin images collected by hand-held devices.

Figure 6 outlines the key processes involved in the automated measurement framework for assessing the biophysical properties of pumpkins.

#### 5.1.1. Overall Description

Detailed explanations for each process are provided as follows.

- Data collection and preprocessing (Figure 6a): The pumpkin component segmentation dataset was collected by a Samsung Galaxy S22 device on two pumpkin green houses in November 2022. The constantly changing light conditions of outdoor environments can lead to inconsistencies in the color of images taken at different times. To address this issue and ensure the quality of the dataset, color correction was performed before the training process.
- Pumpkin segmentation (Figure 6b): SOLOv2 [29] is an extension of the mask region-based convolutional neural network (Mask R-CNN) architecture and enables the identification and localization of individual objects within an image. Unlike the traditional two-stage approach, SOLOv2 utilizes a single-stage network for object detection and segmentation, resulting in faster processing times while maintaining high accuracy. In this study, SOLOv2 is applied to segment the pumpkin’s components accurately.
- Biophysical properties measurement (Figure 6c): This study proposes an automated pipeline that utilizes various CV techniques to measure the real-life values of diverse biophysical properties of pumpkins. The pipeline includes recognizing the ruler positioned near the pumpkin as a reference for measurement. This approach enables the efficient and accurate extraction of biophysical property measurements from the captured images.

#### 5.1.2. Data Preprocessing

The captured images were subjected to color correction to mitigate the impact of varying lighting conditions [23]. In this study, pumpkin color is considered as one of the biophysical properties, making color correction essential to ensure accurate and consistent colors across different images. The color correction was achieved by utilizing a color checkerboard containing a range of color patches with known values. By capturing an image of the checkerboard using the same device, a comparison can be made between the device’s color response and the known values of the patches. This facilitated color adjustments to align the device’s colors with the known values.

The conventional approach for color correction involves selecting a reference image with known color values, which serves as the basis for calculating the color correction matrix [30]. This matrix is then applied to other images, modifying their color channels. By performing matrix multiplication on the red, green, blue (RGB) color channels of the input image using the estimated color correction matrix, the color balance of the image is adjusted to match that of the reference image. This alignment enhances the precision of color representation, providing a more accurate depiction of the actual plant colors. This level of color accuracy is particularly valuable in plant phenotyping applications. The color correction equation can be described as follows:

(1) C=M∗I

where the output image after applying color correction is denoted as *C*. The computed color correction matrix based on the reference image is represented as *M*, and the input image is denoted as *I*.

#### 5.1.3. Pumpkin Segmentation

**Figure 7:**
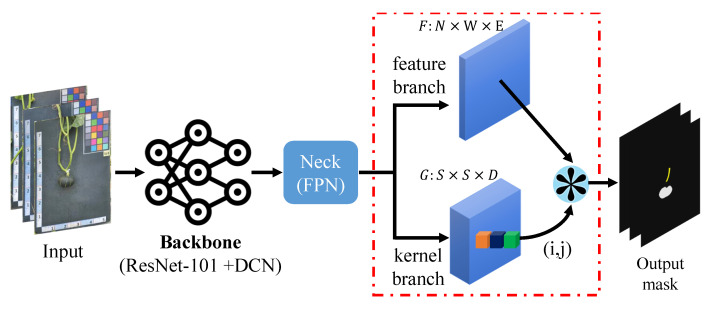
Full architecture of the SOLOv2-based pumpkin segmentation model. Note: *F* represents the feature maps that are extracted from the input image by the backbone network. *N* is the number of feature maps, and *W* is the width and height of the feature maps. *G* represents the grid maps that are used to divide the feature maps into smaller regions, each of which is assigned a separate mask prediction. *S* is the number of grids along each dimension, and *D* is the number of channels in the grid maps.

The SOLOv2 model is considered a state-of-the-art instance segmentation model [31], which is capable of detecting and segmenting multiple objects within an image. Unlike other segmentation models that only predict bounding boxes, SOLOv2 goes a step further by providing pixel-wise instance masks for each object [29]. This unique characteristic makes it a powerful tool with various applications in CV [32,33]. The choice of the SOLOv2 algorithm for pumpkin recognition in this study was driven by its distinct capabilities in precise object segmentation with fast inference speed [34]. This characteristic makes it particularly suitable for our goal of accurately segmenting the different components of pumpkins, namely the vines and fruits. The architecture of the SOLOv2 model is depicted in Figure 7.

The SOLOv2 architecture is built upon a fully convolutional neural network (FCN) [35], which can be represented by the following equation:

(2) $$M_{i}=F_{mask}\left(F_{fusion}\left(F_{backbone}\left(I\right)\right)\right)$$

In this equation, *I* represents the input image, $F_{backbone}$ refers to the backbone network responsible for extracting feature maps from the input image, $F_{fusion}$ denotes the feature fusion module that combines features of different scales and resolutions, and $F_{mask}$ represents the mask prediction module responsible for generating pixel-wise instance masks for each object in the image.

To achieve feature fusion, the feature fusion module utilizes lateral connections that connect the feature maps from various layers of the backbone network to their corresponding layers. These lateral connections can be described using the following equation:

(3) $$P_{i}=Lateral_{i}\left(F_{backbone}\left(I\right)\right)$$

where $Lateral_{i}$ denotes the lateral connection function that connects the feature maps from the *i*-th layer of the backbone network to the corresponding layer in the feature fusion module. In SOLOv2, deformable convolutional networks (DCNs) are employed to replace the standard convolutional layers in the backbone network, allowing for improved handling of the scale variation exhibited by objects in the input images.

The mask prediction module plays a crucial role in generating pixel-wise instance masks by associating each pixel in the image with a specific object instance. This association is established through the use of an embedding vector assigned to each pixel, which determines the object instance to which the pixel belongs. The embedding vector can be mathematically represented as follows:

(4) $$E_{i,j}=F_{embed}{\left(F_{fusion}\left(F_{backbone}\left(I\right)\right)\right)}_{i,j}$$

where $F_{embed}$ represents the embedding function responsible for generating the embedding vectors, and *i* and *j* denote the spatial coordinates of the pixel within the feature maps. Subsequently, the instance masks are generated by assigning each pixel to the object instance with the highest value in the corresponding embedding vector. This process can be expressed as:

(5) $$M_{i,j}=argmax_{k}\left(E_{k,i,j}\right)$$

where *k* is the index of the object instance.

The SOLOv2 model has demonstrated exceptional performance on various benchmark datasets, including common objects in context (COCO), which is widely used for object detection and segmentation [36]. In comparison to other instance segmentation models like Mask R-CNN, SOLOv2 consistently achieves superior results across a range of evaluation metrics. Moreover, the model exhibits high efficiency, and it is characterized by a relatively low parameter count and fast inference speed, making it well-suited for real-world applications [34].

#### 5.1.4. Biophysical Properties Measurement

**Figure 8:**
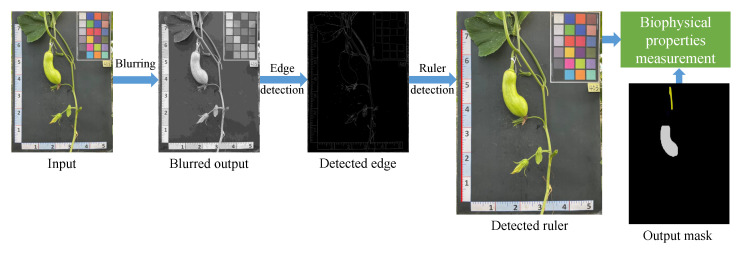
Four main steps of the proposed real-life biophysical properties measurement based on detecting the ruler.

This section presents an automated pipeline designed to accurately measure the physiological properties of various components of pumpkins by detecting the ruler placed alongside the pumpkins in the captured images. The biophysical properties of both the pumpkin fruit and vine were analyzed using a set of one qualitative and four quantitative parameters, adhering to the guidelines provided by the International Union for the Protection of New Varieties of Plants (UPOV, 2021) [37]. The process involves a series of CV operations, as depicted in Figure 8, which includes ruler detection, pixel density conversion, and actual measurement calculation.

The RGB input was initially converted to grayscale to facilitate the identification of edge features [38]. To enhance the image quality and minimize unwanted artifacts that could affect the vertical line detection process, a Gaussian blur was applied to the grayscale image. Subsequently, the Canny edge algorithm, known for its effectiveness in detecting various types of edges while minimizing false detections, was utilized to perform edge detection on the blurred grayscale image. Finally, a Hough line transform technique was employed to identify the ruler based on the detected edges. The resulting straight line (x1,y1,x2,y2) represents the coordinates of the starting (x1,y1) and ending (x2,y2) points of the ruler in the image.

**Figure 9:**
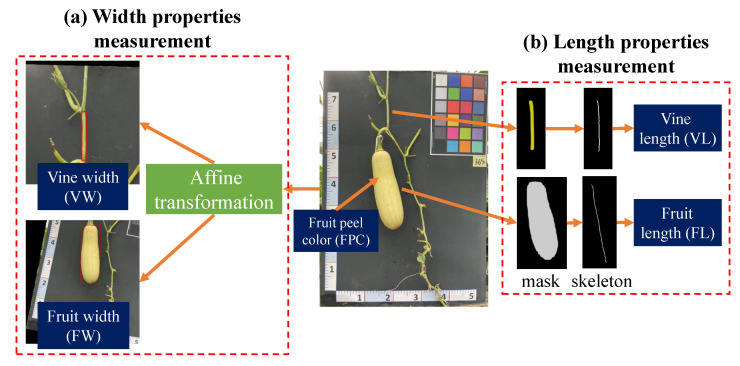
Automated measurement of different pumpkins’ biophysical properties, including VL, VW, FL, FW, and FPC.

Figure 9 presents an overview of the analyzed properties, which include both quantitative and qualitative aspects. The quantitative properties consist of fruit length (FL), fruit width (FW), vine length (VL), and vine width (VW), while the qualitative property is represented by fruit peel color (FPC). In the case of symmetric fruit, the width and length properties can be accurately determined using the bounding box. However, in reality, pumpkin fruits are often captured in non-symmetric orientations, as depicted in Figure 9.

To address this challenge and provide a more precise and comprehensive measurement of the fruit’s biophysical properties, this study proposes an end-to-end approach that takes into account irregularities and asymmetries that may arise during fruit capture. By considering these factors, the proposed approach aims to enhance the accuracy of measurements and enable a more detailed analysis of the fruit’s physical characteristics. This, in turn, holds significant implications for crop management and breeding programs, as it enables a more nuanced understanding of the fruit’s attributes.

**Figure 10:**
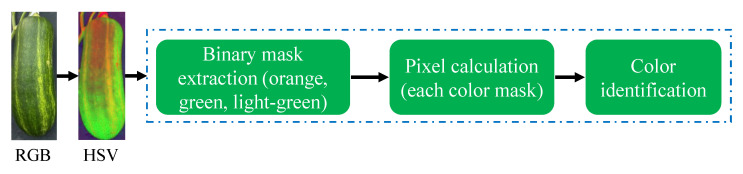
FPC estimation process based on the HSV color channel.

- Width properties measurement (Figure 9a): When dealing with objects that do not have symmetrical shapes, an affine transformation method provides an effective approach to accurately calculate their width, as demonstrated in Figure 9a [39]. Initially, an ellipse is utilized to fit the pumpkin’s fruit and vine components, as it offers a better approximation of the shape compared to a rectangular bounding box. The center coordinates, major and minor axis lengths, and rotation angle of the best-fit ellipse are then extracted, enabling the construction of an affine transformation matrix for rotation. By applying this transformation, the object is aligned with the image’s *x* and *y* axes, facilitating the measurement of width by determining the distance between the two farthest points within the transformed object.
- Length properties measurement (Figure 9b): In order to precisely compute the length property, this study proposes the use of a skeletonization algorithm applied to the segmented mask. This approach offers a more accurate and precise measurement of the length, taking into account any irregularities or asymmetries that may be present in the shape of the fruit. Additionally, the skeleton, being a simplified representation of the fruit’s shape, enables more efficient data processing, facilitating the analysis of large datasets of fruit images.
  The skeletonization process involves iteratively thinning the object or shape until a one-pixel-wide skeleton is obtained. This simplified representation captures the essential features and structure of the object, providing a streamlined depiction of the object [40]. One commonly used method for generating the skeleton is the medial axis transform, which calculates the centerline of the object and produces a skeleton that represents its main axis of symmetry [41]. Figure 9a displays the skeleton output obtained from the medial axis skeletonization method applied to the input vine and fruit masks. The resulting image is binary, with pixels on the skeleton assigned a value of 1, while all other pixels are assigned a value of 0.
  Previous research has shown that the object length can be determined using the following formula once the object skeleton is extracted [8]:
  (6) $$Length=\int_{c}Cdl≅\sum Cdl$$
  where the finite length of *L* is denoted by dl, and *C* represents the geometric calibration. Initially, *C* was introduced as a calibration factor to account for pixel displacements in the mask outputs. However, since the dataset used in this study exhibited no geometric distortion, the parameter *C* was set to 1. This allowed for the direct summation of the total pixels along the skeleton to calculate *L*.
- Color estimation: The estimation of the pumpkin’s FPC was based on the standard color variations observed in pumpkins. According to findings of Kaur et al. [1], the color of the pumpkin fruit peel can exhibit variations based on factors such as the pumpkin variety and maturity stage. Typically, pumpkin fruit peel is characterized by an orange hue, which can range from a pale, light orange to a deep, rich shade. Additionally, certain pumpkin varieties may feature green, yellow, or white stripes or patches on their peel. As the pumpkin undergoes ripening, the peel color tends to deepen and become more vibrant. Hence, this study focused on three main FPC categories: orange, green, and light green.
  Figure 10 visually presents the process of classifying pumpkin’s FPC using the hue, saturation, and value (HSV) color space. The HSV color space is favored over RGB for color detection tasks due to its ability to separate color information from brightness or luminance information, thereby providing a more intuitive representation [42]. In this process, specific color ranges within the HSV color space were defined, enabling the creation of binary masks for each color range. These binary masks were then employed to determine the FPC by identifying the color range with the highest pixel count.

### 5.2. Experimental Settings

#### 5.2.1. Hardware and Software Platform

The proposed framework for measuring the biophysical properties of pumpkins was primarily developed using Python 3.7 on a Ubuntu 20.04 system equipped with two Nvidia Tesla V100 GPUs, each with 32 gigabytes of memory. The SOLOv2 segmentation model was constructed using MMdetection 3.1.0 [43], an open-source object detection framework based on PyTorch 2.0.0. In addition, PlantCV 1.1 [44], an open-source Python library specifically designed for plant image analysis was implemented in this study. PlantCV offers comprehensive support for a range of tasks, including feature extraction, image processing, and data analysis.

#### 5.2.2. Optimizer, Loss Function and Hyperparameters

The SOLOv2-based model used a pre-trained ResNet-101, which was initially trained on the ImageNet dataset [45], as its backbone. During training, the model was specifically configured to detect two classes: fruit and vine. The intermediate feature maps, consisting of 512 channels, were utilized for generating precise object masks. The mask head of the SOLOv2 model incorporated four convolutional layers to refine the mask predictions. To optimize the model’s performance in generating accurate object masks, the dice loss function was employed as the primary loss function during the training process. The model was trained for 25 epochs with an Adam optimizer, using an initial learning rate of $e^{-3}$ and weight decay of $e^{-4}$. The mini-batch size was set to 4.

#### 5.2.3. Evaluation Metrics

Mean Average Precision (mAP) is a widely adopted evaluation metric for assessing the performance of segmentation models on the COCO segmentation dataset [36]. The evaluation process involves sorting the model’s predicted segmentation masks based on their confidence scores. Precision and recall values are then computed for each class label at various confidence thresholds. Precision represents the fraction of predicted pixels for a given class that have an Intersection over Union (IoU) greater than the threshold, while recall represents the fraction of ground truth (GT) pixels for that class with an IoU greater than the threshold. By constructing a precision–recall curve, the Average Precision (AP) can be calculated. Finally, the mAP is obtained by averaging the AP values across all class labels in the dataset. The equation for mAP is expressed as follows:

(7) $$mAP=\frac{1}{C}\sum_{c=1}^{C}AP_{c}$$

where *C* is the number of semantic classes in the dataset and $AP_{c}$ is the average precision for class *c*.

To assess the predictive capability of the model in capturing various physiological properties of pumpkins, two additional evaluation metrics, namely mean absolute error (MAE) and mean absolute percentage error (MAPE) [46], are computed.

MAE calculates the mean absolute difference between the the GT values and the predicted values, presenting a numerical measure of the model’s error magnitude. A lower MAE signifies superior performance by indicating a smaller average deviation between the predicted and GT values. With a different technique, MAPE calculates the average percentage difference between the GT values and the predicted values. It represents the errors as a percentage of the actual values, providing a relative assessment of the model’s performance. MAPE is particularly valuable when substantial variations in data scale or magnitude occur across different samples. Like MAE, a lower MAPE denotes better performance by indicating reduced percentage errors between the predicted and actual values. The equations for *MAE* and *MAPE* are defined as follows:

(8) $$MAE=\frac{1}{N}\sum_{i=1}^{N}\left|y_{i}-{\hat{y}}_{i}\right|$$

(9) $$MAPE=\frac{1}{N}\sum_{i=1}^{N}\left|\frac{y_{i}-{\hat{y}}_{i}}{y_{i}}\right|\times 100$$

where *N* represents the number of biophysical properties, $y_{i}$ indicates the GT physiological property value, and ${\hat{y}}_{i}$ is the predicted physiological property value. The absolute value |.| is used to ensure that the errors are positive values.

## 6. Conclusions and Future Works

This study presents the development of an automated framework specifically designed for measuring the biophysical properties of pumpkins, with potential applications in breeding selection programs. To facilitate accurate analysis, a comprehensive dataset consisting of 900 high-resolution images was collected, covering two pumpkin varieties, namely *C. moschata* and *C. maxima*.

The framework incorporates several crucial modules. Firstly, a color correction technique was implemented to ensure consistent and accurate color representation across all images in the dataset. Compared to other well-known segmentation models (BlendMask, HTC, MS R-CNN, and Mask-RCNN), the SOLOv2-based model demonstrated the highest validation mAP of 88% and the fastest inference speed of 19.4 FPS, enabling precise segmentation of the vine and fruit components of the pumpkin. Furthermore, the framework employed the extraction of the fruit’s skeleton for measuring the length trait and an affine transformation method for accurately determining the width trait. These techniques contributed to achieving highly accurate measurements compared to manual methods with an MAPE of about 2.5%.

Although this study mainly focused on the biophysical properties of pumpkins, the framework has the potential to be applied to other plants, such as cucumbers and radishes, given similar settings and sufficient segmentation datasets. The establishment of a measurement standard for the biophysical properties of pumpkin to guide output analysis would also be an intriguing topic for future exploration. Furthermore, since the current framework is not suitable for real-time measurements due to its complexity, future work should prioritize optimizing the framework for robustness and time efficiency to enable real-time measurement capabilities.

## Figures and Tables

**Table 1:** Comparison of different segmentation algorithms on the collected pumpkin dataset.

Model	Mask AP (%)	Inference Speed (FPS)
BlendMask [18]	85.9	11
HTC [19]	85.6	6
MS R-CNN [20]	82.1	10.7
Mask-RCNN [21]	84.8	11.2
SOLOv2	**88**	**19.4**

Note: The optimal values for each metric are highlighted in bold font. HTC refers to the hybrid task cascade model, MS R-CNN denotes the mask scoring R-CNN model, and SOLOv2 represents scalable object localization with a balanced positive instances-based model. AP stands for average precision, which serves as an evaluation metric, and FPS represents frames per second, which quantifies the inference speed.

**Table 2:** Comparison between the GT and the predicted biophysical properties measurement for 10 different pumpkin samples.

		Sample									
		S1	S2	S3	S4	S5	S6	S7	S8	S9	S10
GT	FL (mm)	6.8	27.8	14.1	8.4	9.5	22.6	25.9	6.2	6.9	6.3
	FW (mm)	10.2	9.6	12.2	12.9	12.1	9.2	8.1	8.6	11.1	9.7
	VL (mm)	12.2	10.3	16.2	12.8	10.7	10	8.9	14.8	10.6	6.5
	VW (mm)	1.4	0.9	1.2	1.64	1.8	0.67	1	1.1	1.4	1.6
	FPC	G	OR	LG	G	G	LG	LG	G	G	G
Pre	FL (mm)	6.8	28.1	14.1	8.3	9.2	23	26.1	6.5	7	6.8
	FW (mm)	9.9	9.1	12.3	13.1	12	9.1	8	8.9	11.5	9.5
	VL (mm)	12	10.1	15.9	12.6	10.9	9.8	8.4	14.2	10	6.3
	VW (mm)	1.4	0.85	1.3	1.63	1.8	0.68	1	1.2	1.3	1.6
	FPC	G	OR	LG	G	G	LG	LG	G	G	G
Accuracy (%)		99.7	98.6	100	82.9	100	93.7	96.1	97.4	99	96.1
MAE		0.12	0.26	0.12	0.15	0.15	0.2	0.2	0.32	0.3	0.22
MAPE (%)		1.14	4.4	2.75	2.6	1.46	1.58	1.9	1.9	4.46	3.2

Note: S1–S10 represent the pumpkin samples numbered from 1 to 10. GT denotes the ground truth values manually measured, while Pre refers to the predicted values. FL represents the fruit length, FW denotes the fruit width, VL stands for vine length, VW represents vine width, and FPC represents fruit peel color. G stands for green, OR represents orange, and LG indicates light green color. Additionally, MAE refers to the mean absolute error, and MAPE signifies the mean absolute percentage error.

## Data Availability

Data available on request due to restrictions, e.g., privacy or ethical.

## Abbreviations

The following abbreviations are used in this manuscript:

RGB	Red–green–blue color channel
RCNN	Region-based convolutional neural network
UAV	Unmanned aerial vehicle
ISSR	Inter-simple sequence repeat
FPN	Feature pyramid network
FL	Fruit length
FW	Fruit width
VL	Vine length
VW	Vine width
FPC	Fruit peel color
HSV	Hue, saturation, and value
CNN	Convolutional neural network
DCN	Deformable convolutional networks
